# Metformin protects against mouse oocyte apoptosis defects induced by arecoline

**DOI:** 10.1111/cpr.12809

**Published:** 2020-06-17

**Authors:** Wei‐Dong Li, Chuan‐jie Zang, Shen Yin, Wei Shen, Qing‐Yuan Sun, Minghui Zhao

**Affiliations:** ^1^ College of Animal Science and Technology Qingdao Agricultural University Qingdao China; ^2^ College of Life Sciences Institute of Reproductive Sciences Qingdao Agricultural University Qingdao China; ^3^ State Key Laboratory of Stem Cell and Reproductive Biology Institute of Zoology Chinese Academy of Sciences Beijing China

**Keywords:** apoptosis, arecoline, cytoskeleton, metformin, mitochondrion, oocyte

## Abstract

**Objectives:**

Arecoline is the main bioactive substance extracted from Areca catechu L, which has cell, neural and genetic toxicity. The function of arecoline in reproductive system has not been well explored.

**Materials and Methods:**

To investigate the toxic effects of arecoline on oocyte development, immunofluorescence staining, qPCR, Western blotting, sperm binding assays and in vitro fertilization were performed to evaluate oocyte meiosis competence and embryo development.

**Results:**

Our data revealed that arecoline exposure disrupts actin filament dynamics, spindle assembly and kinetochore‐microtubule attachment stability in mouse oocytes, leading to aneuploidy and oocyte meiosis arrest. In addition, arecoline treatment disturbs the distribution of mitochondria, reduces ATP production and increases the level of oxidative stress, which ultimately induces oocyte apoptosis. Supplementation with metformin, a medicine for type 2 diabetes in the clinic, partially alleviates these damages.

**Conclusions:**

Metformin has a protective effect on arecoline‐induced mouse oocytes apoptosis.

1 **HIGHLIGHTS**

- Improvement effects of metformin on arecoline‐exposed mouse oocytes.
- Metformin protects the cytoskeletal integrality and euploidy.
- Metformin restores mitochondrial dysfunctions.

## 1 INTRODUCTION

*Areca catechu* L. (Arecaceae) is widely distributed in East Asia, South Asia and Southeast Asia such as China, India, Indonesia, Malaysia and the Philippines.^1^ It is the fourth most common class of psychoactive substances in the world following tobacco, alcohol and caffeine.^2^ According to statistics, about 600 million people around the world have the habit of chewing betel nut, accounting for about one‐tenth of the world's total population,^3^, ^4^ and at least 400 million people chew betel nuts every day.^5^, ^6^ Based on hundreds of research reports in seven countries, International Agency for Research on Cancer (IARC) officially identified betel nut as a primary carcinogen in 2003.^7^

Arecoline (1,2,5,6‐tetrahydro‐1‐methyl‐3‐pyridinecarboxylic acid methyl ester) is an alkaloid isolated from betel nut and is considered as the main active ingredient of betel nut.^8^ Arecoline has not only cytotoxicity,^9^, ^10^ neurotoxicity,^11^ genotoxicity^12^ and immunotoxicity,^13^ but also reproductive toxicity in male.^14^, ^15^ According to the survey, pregnant women who chew betel nut for a long time will experience symptoms such as decreased fertility, premature birth and miscarriage, and most of the babies they give are characterized by underweight and stunting.^16^, ^17^, ^18^ Studies have shown that exposure to arecoline significantly reduced the hatching rate and survival rate of zebrafish embryos, resulting in embryonic development retardation.^19^, ^20^ At the same time, arecoline also reduces the formation of implanted embryos in the early stages of pregnancy and inhibits the growth of blastocyst trophoblast cells.^21^ In addition, arecoline can also cause a decrease in the number of spermatozoa in the epididymis, a decrease in sperm motility and a malformation in sperm morphology.^14^, ^15^ However, the toxic effects of arecoline on oocyte maturation and its specific mechanisms remain unknown.

The mammalian oocytes originate from primordial germ cells, undergo complex processes of mitosis and meiotic maturation and then arrest at the MII stage. Only after fertilization or parthenogenetic activation, the oocytes initiate the early embryonic development.^22^ During the first meiotic process, with the germinal vesicle breakdown, the chromosome is directly exposed to the cytoplasm of the oocytes, opening a very sensitive time window when chemical substances, such as toxins or drugs, can cause the loss of nuclear and cytoplasmic maturation, affecting the development potential.^22^ Exposure to chemicals can significantly induce excessive reactive oxygen species (ROS) production^23^ causing mitochondrial dysfunction,^24^ resulting in disruption of cytoskeletal integrality and early apoptosis, which can lead to poor embryo quality and delay or even prevent embryo development.^25^ It is worth noting that mitochondrial control of apoptosis has been described at several levels, including ATP production,^26^ mitochondrial membrane potential and mitochondrial membrane permeability to apoptogenic factors, which can be released from the intermembrane space into the cytosol.^27^

Metformin is recommended by experts from the American diabetes association and the European diabetes research association as a first‐line treatment for type 2 diabetes.^28^ The study found that in addition to hypoglycaemic effects, metformin is also playing an active role in fighting cancer,^29^, ^30^, ^31^, ^32^ delaying ageing^33^, ^34^, ^35^, ^36^ and regulating immunity.^37^, ^38^, ^39^ At the same time, numerous studies have pointed out that metformin plays an important role in the reproductive system.^40^, ^41^, ^42^ On the one hand, metformin treatment not only reduces oxidative stress and germ cell loss, thereby limiting spermatogenesis damage, but also increases the number and viability of rat spermatozoa.^43^ On the other hand, metformin can reverse ovulation dysfunction and improve oocyte quality and embryo development in mice with polycystic ovary syndrome (PCOS) by reducing the level of ROS in oocytes and improving mitochondrial function.^44^ In addition, studies in embryos have shown that metformin can also reduce apoptosis in blastocysts of obese mice.^45^ Although the reproductive safety of metformin is still unclear, in general, metformin helps restore fertility and contributes significantly to the maturity and embryonic development of high‐quality female germ cells.

To date, there have been no reports of the effects of arecoline on oocyte maturation. In this study, we explored the toxic effects of arecoline on the quality of cultured mouse oocytes and the rescue function of metformin. The results showed that arecoline leads to disordered progression of oocyte meiosis and increased ROS by destroying cytoskeletal integrity, chromosome euploidy and mitochondrial function, eventually leading to oocyte apoptosis. However, metformin could partially reduce these damages, suggesting that metformin has a protective effect against the apoptosis of oocytes induced by arecoline.

## 2 MATERIALS AND METHODS

### 2.1 Ethics statement

All the experimental procedures in this study strictly implemented the standards of the Ethics Committee of Qingdao Agricultural University. Institute of cancer research (ICR) female mice were fed a regular diet in a temperature‐controlled room of 12 hours light/dark cycle. In order to alleviate the suffering as much as possible, the mice were sacrificed humanely by the cervical dislocation during the collection of the oocytes.

### 2.2 Oocyte collection and culture

The collection of oocytes was performed as described previously.^22^ Briefly, after 48 hours of treatment with pregnant mare serum gonadotropin (PMSG), 4‐ to 6‐week old ICR female mice were euthanized and ovaries were collected in M2 medium supplemented with 2.5 μmol/L milrinone. The morphologically intact cumulus‐oocyte complexes (COCs) were picked up by a pipette under a stereomicroscope and washed 3‐5 times in M2 operating droplets. Each set of 25‐30 oocytes in germinal vesicle (GV) stage were placed in approximately 30 μL of M16 medium droplets covered with mineral oil and in vitro matured at 37°C in 5% humidified CO_2_ atmosphere.

### 2.3 Arecoline and metformin treatment

Arecoline (98%, 252 164; J&K Chemical Ltd., Beijing, China) was dissolved in ultrapure water as stock solution and diluted in the M16 medium as working solution. Metformin (98%, 257 998; J&K Chemical Ltd., Beijing, China) was dissolved in double distilled water (ddH_2_O) as stock solution and diluted in the M16 medium as working solution. In order to test effects of arecoline exposure on oocyte meiosis progression, oocytes were matured in M16 medium supplement with different concentration of arecoline with or without metformin, according to experiment design. For detection of maturation rate, the 1st polar bodies were evaluated at 12 hours of in vitro culture. According to the results, we selected 180 μg/mL arecoline and 50 μmol/L metformin for the following experiments because this concentration not only caused meiotic arrest but also allowed proper number of oocytes to develop into the MII maturation stage (~50%) for other investigations.

### 2.4 Immunofluorescence and confocal microscopy

The oocytes were first fixed in 4% paraformaldehyde (PFA) for about 30 minutes at room temperature (RT). After several washes, the oocytes were permeabilized for 20 minutes in Dulbecco's phosphate‐buffered saline (D‐PBS) supplemented with 0.5% Triton X‐100 and blocked for 1 hour in D‐PBS containing 1% bovine serum albumin (BSA). The oocytes were then incubated in the appropriate primary antibody at 4°C overnight. Next, the oocytes were incubated for 1 hour at RT in the corresponding secondary antibody. The DNA was counterstained with 2‐(4‐Amidinophenyl)‐6‐indolecarbamidine dihydrochloride (DAPI, C1022; Beyotime Institute of Biotechnology, Shanghai, China) for about 20 minutes. After washing, they were installed on glass slides with a little fluorescent anti‐quenching agent. Representative images were captured by using a laser‐scanning confocal microscope (Leica TCS SP5 II, Mannheim, Germany). Among the primary antibodies include anti‐centromere CREST antibody (1:200; 15‐234‐0001; Antibodies Incorporated, CA), anti‐α‐tubulin antibody (1:500; SC‐5286; Santa Cruz Biotechnology, TX) and Phalloidin (1:1000; 23 122; AAT Bioquest, CA).

### 2.5 RNA extraction and quantitative real‐time PCR (qRT‐PCR)

In accordance with the instructions, about 30 oocytes were collected and messenger RNA (mRNA) was extracted using Dynabeads mRNA DIRECT Kit (Thermo Fisher scientific Inc, Waltham, MA, USA). HiScript II Q RT SuperMix (Vazyme Biotech Co. Ltd, Nanjing, China) was used for the generation of first‐strand cDNA accord to the instruction. And AceQ qPCR SYBR Green Master Mix (Vazyme Biotech Co. Ltd, Nanjing, China) was used for qRT‐PCR in an ABI 7500 Sequence Detection System. Ten pairs of primers are listed in the Table 1. Amplification was performed in 20 μL reactions containing 10 μL of SYBR Green Master Mix, 7.2 μL of RNase‐free H_2_O, 0.4 μL of primers (10 μmol/L), 0.4 μL of ROX Reference Dye2 and 2 μL of cDNA. The amplification conditions of PCR were as follows: reaction was initialled at 95°C for 5 mins, followed by 40 cycles of denaturing at 95°C for 10 s, annealing at 60°C for 34 s and extension at 72°C for 20 s. The 2^−△△Ct^ method was used to calculate the relative gene expression to *β‐actin* or *gapdh*.

**Table 1:** Primers and amplification conditions used for qRT‐PCR

Genes	Accession (NCBI)	Primer sequences	Size(bp)	Temperature (°C)
*Cat*	NM_009804.2	F: CAGCGACCAGATGAAGCAGT R: CCTCAAAGTATCCAAAAGCACC	236	59
*Gpx1*	NM_001329528.1	F: GGAGAATGGCAAGAATGAAGA R: AGGAAGGTAAAGAGCGGGTG	135	58
*Prdx2*	NM_011563.6	F: GATGGTGCCTTCAAGGAAATCA R: CCGTGGGGCAAACAAAAGTG	97	60
*Prdx6*	NM_001303408.1	F: CGCCAGAGTTTGCCAAGAG R: TCCGTGGGTGTTTCACCATTG	115	60
*Sod1*	NM_011434.1	F: GGGTTCCACGTCCATCAGTA R: TTGCCCAGGTCTCCAACAT	128	59
*Sod2*	NM_013671.3	F: CAGACCTGCCTTACGACTATGG R: CTCGGTGGCGTTGAGATTGTT	113	61
*Bax*	NM_007527.3	F: ATGCGTCCACCAAGAAGCTGAG R: CCCCAGTTGAAGTTGCCATCAG	166	62
*Bak*	NM_007523.3	F: CAACCCCGAGATGGACAACTT R: CGTAGCGCCGGTTAATATCAT	101	59
*Caspase3*	NM_001284409.1	F: GACTGGGATGAACCACGACCC R: TCTGACTGGAAAGCCGAAAC	205	60
*Caspase9*	NM_015733.5	F: CTGGGAAGGTGGAGTAGGAC R: GCGGTGGTGAGCAG	189	56
*β‐actin*	NM_007393.5	F: TCGTGGGCCGCCCTAGGCAC R: TGGCCTTAGGGTTCAGGGGGG	243	59
*Gapdh*	NM_001289726.1	F: GACAAAATGGTGAAGGTCGGT R: GAGGTCAATGAAGGGGTCG	120	58

### 2.6 Western blotting analysis

About 100 oocytes were lysed in the RIPA buffer supplemented with the protease inhibitor cocktail from Beyotime Institute of Biotechnology (P0013B and P1006; Shanghai, China). The target protein was first separated by sodium dodecyl sulphate‐polyacrylamide gel electrophoresis (SDS‐PAGE) and then transferred to a polyvinylidene fluoride (PVDF) membrane. Next, the membrane was blocked in TBST containing 5% BSA for 1 hour at room temperature, followed by incubation overnight at 4°C with anti‐CAT rabbit polyclonal antibody (1:200), anti‐BAX rabbit polyclonal antibody (1:500) and anti‐ACTB rabbit polyclonal antibody (1:1000). After washing 3‐5 times in TBST for 5‐10 minutes, the membrane was incubated with HRP‐conjugated goat anti‐rabbit IgG (1:2000) for 1 hour at 37°C in the dark. All four antibodies were purchased from Sangon Biotech Co., Ltd. (D122036, D120073, D110001 and D110058; Shanghai, China).

### 2.7 Parthenogenetic activation

Due to extremely low efficiency of fertilization in vitro using in vitro matured oocytes, the parthenogenetic activation was employed for evaluation of oocytes quality. After extruding the first polar body, 25‐30 oocytes were collected and treated in pre‐equilibrated Ca^2+^‐free Chatot, Ziomek, Bavister (CZB) medium containing 10 mmol/L SrCl_2_ (A500908; Sangon Biotech, Shanghai, China) for 4‐6 hours at 37°C. The hallmark of successful activation of oocytes is the appearance of pronucleus.

### 2.8 Sperm binding assay

Cauda epididymal spermatozoon was isolated from male mice and then placed in human tubal fluid (HTF) medium under mineral oil, which was previously equilibrated to a density of 1 × 10^6^ cells/mL at 37°C, 5% CO_2_ atmosphere_._ After 1 hour of sperm capacitation, 20 μL of the capacitated sperm solution was added to the HTF medium containing oocytes, and the final sperm concentration was 5 × 10^5^ cells/mL, followed by incubation for 2 hours. The sperm‐oocyte combinations were fixed in 4% PFA for 30 minutes and then stained with DAPI.

### 2.9 Chromosome spread

The MII stage eggs were first placed in an acidic Tyrode solution (pH = 2.5) for a few seconds. After removing the zona pellucida, the oocytes were transferred to M2 medium as quickly as possible and washed for several times. Then, 25‐30 oocytes were transferred to a glass slide and fixed in an alkaline solution of 1% PFA (pH = 9.2) in distilled water containing 0.15% Triton X‐100 and 3 mmol/L dithiothreitol. After the air was naturally dried, the chromosome was counterstained with propidium iodide (PI, E607306; Sangon Biotech, Shanghai, China) and visualized by laser‐scanning confocal microscopy.

### 2.10 Mitochondrial distribution

Mitochondria were stained by using MitoTracker Deep Red (M22426; Invitrogen, UK) to assess the distribution of mitochondria in oocytes. The oocytes were incubated in a pre‐warmed staining solution for 30 minutes at 37°C. After washing, the oocytes were counterstained with DAPI to visualize the nucleus. The image was observed and captured using a laser‐scanning confocal microscope. Mitochondria distribution manner were separated into three typical groups include homogenous, perinuclear and clustering, according to Sun et al’s description.^46^

### 2.11 Mitochondrial membrane potential (Δψm) assay

To measure mitochondrial membrane potential, oocytes were washed three times with PBS and incubated in culture medium containing 0.5 μmol/L JC‐1 (Invitrogen, Grand Island, NY, USA) at 37°C in 5% CO_2_ for 30 minutes. Membrane potential was calculated as the ratio of red florescence, which corresponded to activated mitochondria (J‐aggregates), to green fluorescence, which corresponded to less‐activated mitochondria (J‐monomers).^47^ Fluorescence was visualized by laser‐scanning confocal microscopy.

### 2.12 Adenosine 5'‐triphosphate (ATP) content detection

Detection of ATP content was followed the manual of the Enhanced ATP Assay Kit (S0027; Beyotime Institute of Biotechnology, Shanghai, China). Briefly, a same number (n = 15) of oocytes obtained from each group were lysed in 4 μL water by ultrasonic, followed by mixed with 16 μL of detection solution. After 15 minutes of reaction, the ATP concentration was measured using a luminometer (Berthold, Wildbad, Germany) with a sensitivity of 0.01 pM. The ATP concentration in the control group was arbitrarily set to 1. Three separate experiments were performed with three replicates in each.

### 2.13 ROS generation detection

Reactive Oxygen Species Assay Kit (S0033; Beyotime Institute of Biotechnology, Shanghai, China) was performed to detect ROS content of oocytes. Concisely described as incubation with dichlorofluorescein diacetate (DCFH‐DA) probe in the dark at 37°C for 20 minutes, after washing, the oocytes were installed on a glass slide and observed under a laser‐scanning confocal microscope with the same scanning parameters for all measurements.

### 2.14 Annexin V staining

Apoptosis detection was performed by using the Annexin V‐FITC Apoptosis Kit (C1063; Beyotime Institute of Biotechnology, Shanghai, China). Oocytes were stained with 5 μL of Annexin V‐FITC diluted in 195 μL of binding buffer for 15 minutes at RT in the dark. After washing, the oocytes were mounted on a glass slide to monitor the fluorescence signal and images were captured by using a laser‐scanning confocal microscope with the same scanning parameters.

### 2.15 Fluorescence intensity analysis

All images were captured using the same scan parameters when performing fluorescence intensity analysis. The average fluorescence intensity per unit area within the region of interest was analysed by using ImageJ software (NIH, Bethesda, MD).

### 2.16 Statistical analysis

Each experiment was repeated at least 3 times, each group involving 25‐30 oocytes. Data were taken as mean ± standard deviation and analysed by one‐way ANOVA using SPSS software. Differences were considered to be statistically significant, *P* < .05.

### 2.17 Experiment design

**Figure 1:**
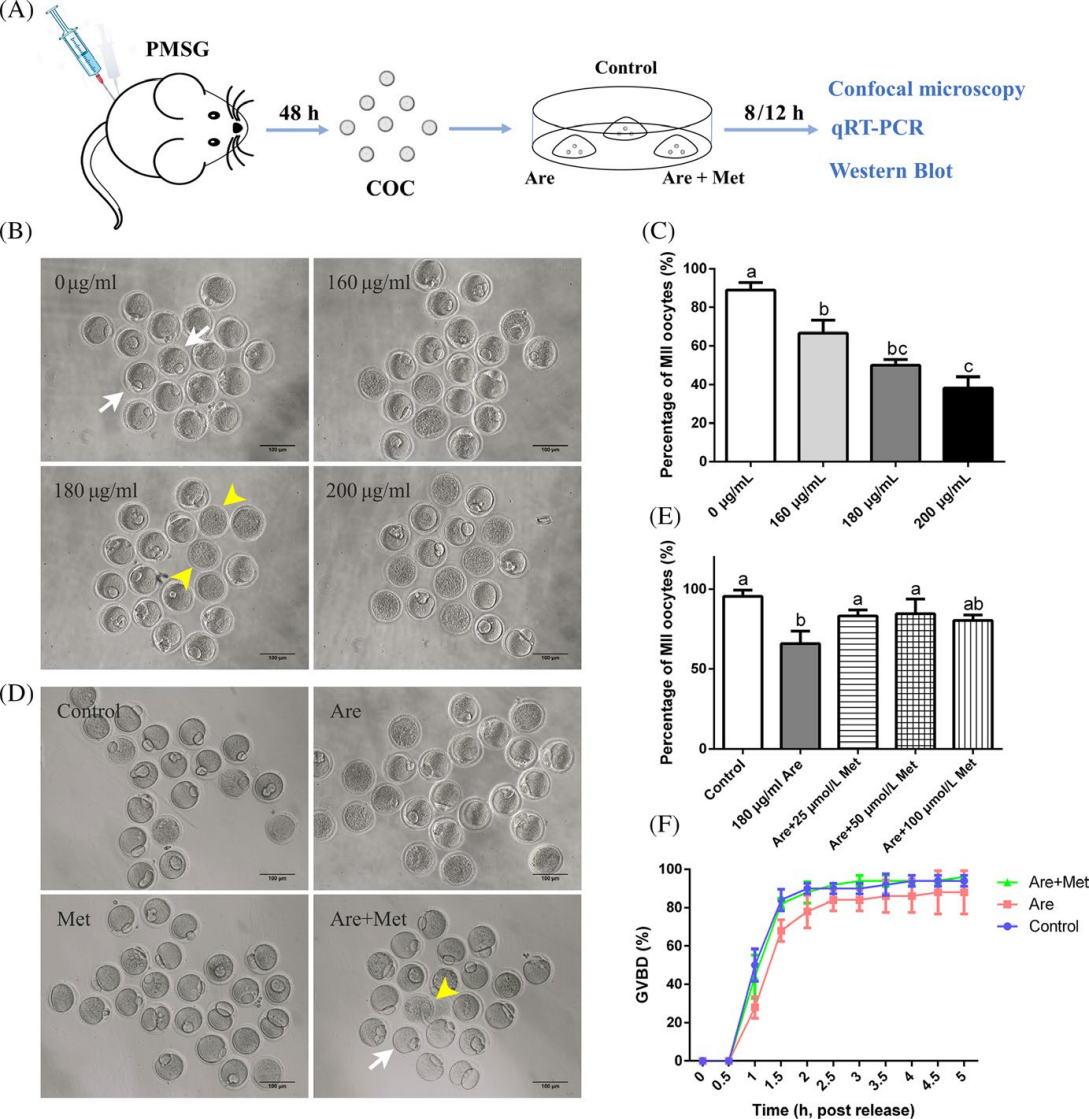
Effects of metformin (Met) on the meiotic maturation in the arecoline (Are)‐exposed oocytes. A, Experimental process overview. B, Representative images of oocytes after 12 h of culture were treated with Are. C, The rates of first polar body extrusion after Are treatment. D, Representative images of oocytes in the control, Are and Are + Met groups. E, The rates of first polar body extrusion in the control, Are and Are + Met groups. F, The rates of germinal vesicle breakdown. Scale bar = 100 μm. White arrow shows the MII stage oocyte with the first polar body, and yellow arrowhead shows the oocyte that has undergone apoptosis. Data were presented as mean ± SD. ^a‐c^Means not sharing a common superscript are different (*P* < .05)

*Experiment 1: Metformin improves the meiotic maturation in arecoline‐exposed oocytes.* To verify whether arecoline toxic to oocytes, as shown in Figure 1A, we obtained COCs from mice 48 hours after PMSG injection, and we counted the first polar body exclusion rate after 12 hours of culture by setting three sets of concentration gradients: 160, 180 and 200 μg/mL. To verify whether metformin has a certain rescue function for the damage caused by arecoline, oocytes were randomly assigned into three groups: control, arecoline and arecoline‐metformin groups. The concentrations of metformin were 25, 50 and 100 μmol/L, respectively. According to the results, we selected 180 μg/mL arecoline and 50 μmol/L metformin for the following experiments.

*Experiment 2: Metformin restores the actin dynamics, spindle defects and chromosome misalignment in arecoline‐exposed oocytes.* The collapsed cytoskeleton was first considered to explain the cause of the first polar body failed to extrude. Therefore, we next evaluated the two most basic oocyte cytoskeletal proteins in actin filaments and spindle microtubules by fluorescence staining.

*Experiment 3: Metformin maintains euploidy by restoring defective kinetochore‐microtubule (K‐M) attachment in arecoline‐exposed oocytes.* In general, the failure of the assembly of the spindle tends to impair the stability of the K‐M attachment, which in turn causes oocyte aneuploidy. Immediately, we studied the co‐localization of spindles, chromosomes and centromeres and the karyotype of oocytes.

*Experiment 4: Metformin elevates the rate of homogeneous mitochondrial distribution, ATP content and mitochondrial membrane potential in arecoline‐exposed oocytes.* To explore the causes of cytoskeletal collapse at a deeper level, we examined the distribution of mitochondria and determined the amount of ATP production, as insufficient energy supply also led to failure of actin filament and spindle assembly. However, the reduction in ATP content is often caused by damage to mitochondria. Therefore, we next analysed the mitochondrial membrane potential of oocytes, a landmark indicator of mitochondrial damage.

*Experiment 5: Metformin reduces ROS level and limits early apoptosis in arecoline‐exposed oocytes.* Mitochondrial dysfunction is often accompanied by the production of large amounts of ROS, which in turn leads to apoptosis in oocytes. To test this hypothesis, we first examined changes in reactive oxygen species and early apoptosis by fluorescence staining. Secondly, in order to further explore the underlying mechanism of apoptosis, we quantified apoptosis‐related genes and proteins, and revealed that arecoline has attenuated oocyte apoptosis by activating the caspase signalling pathway. However, metformin could partially reduce these damages, suggesting that metformin has a protective effect against the apoptosis of oocytes induced by arecoline.

## 3 RESULTS

### 3.1 Metformin improves the meiotic maturation in arecoline‐exposed oocytes

Firstly, we investigated the effects of arecoline exposure on the meiotic progression of mouse oocytes. Increasing concentrations of arecoline (160, 180 and 200 μg/mL) were supplemented to M16 culture medium to detect the first polar body extrusion (PBE). As shown in Figure 1B, most oocytes reached the metaphase II (MII) stage with the extrusion of the polar body in the control group (89.02 ± 3.75%, n = 305). However, arecoline exposure resulted in oocyte meiosis arrest with the prominently decreased PBE (160 μg/mL: 66.67 ± 6.73%, n = 115, *P* < .05, 180 μg/mL: 50.00 ± 3.09%, n = 317, *P* < .01, 200 μg/mL: 38.22 ± 5.79%, n = 117, *P* < .01), which indicated that arecoline exposure inhibits meiotic maturation of mouse oocytes in a dose‐dependent manner (Figure 1C). Based on the results, we chose 180 μg/mL arecoline for subsequent experiments because this concentration not only significantly reduced the maturation rate compared to the control, but also allowed the appropriate number of oocytes to develop into the MII stage for other studies.

To evaluate whether metformin could alleviate the meiotic arrest caused by arecoline, metformin was added into the 180 μg/mL arecoline‐supplemented M16 to yield a final concentration of 25, 50 and 100 μmol/L, respectively. Quantitative analysis revealed that 50 μmol/L metformin increased significantly the PBE (84.69 ± 9.03%, n = 616, *P* < .05) compared with the exclusive treatment with arecoline (Figure 1D and E; 65.83 ± 8.04%, n = 247). Higher concentration of metformin did not visibly promote the PBE rate. According to the results, we selected 50 μmol/L metformin for the further exploration. In addition, we have also detected the germinal vesicle breakdown (GVBD), another key step in the progression of meiosis. The results indicated that although arecoline caused a slight delay in the occurrence of GVBD, there was no statistical difference compared with the control (Figure 1F).

### 3.2 Metformin partially enhances the ability of sperm binding to zona pellucida and parthenogenetic activation in arecoline‐exposed oocytes

The study of sperm binding ability is important for the evaluation of the fertilization potential of oocytes. Thus, we counted the number of sperm binding to the zona pellucida surrounding unfertilized eggs by DAPI staining. As shown in Figure S1A and B (n = 194), although arecoline did not markedly reduce the number of sperm binding to the zona pellucida compared to the control, the metformin‐rescued group significantly elevated the number of bound spermatozoa compared with the arecoline‐exposed group.

Parthenogenetic activation is great method for evaluation of embryo development. Then, we calculated the rate of development to blastocysts after parthenogenetic activation of MII stage eggs. The results showed that blastocyst rate dramatically decreased in arecoline‐exposed group compared with control. However, metformin did not rescue the embryo development in the presence of arecoline (Figure S1C and D; 77.04 ± 6.12%, n = 192 *vs* 43.18 ± 3.35%, n = 219, *P* < .01 *vs.* 52.88 ± 1.18%, n = 210, *P* > .05). Taken together, these observations implied that metformin could improve partially the ability of sperm binding to zona pellucida and parthenogenetic activation.

### 3.3 Metformin restores the actin dynamics in arecoline‐exposed oocytes

**Figure 2:**
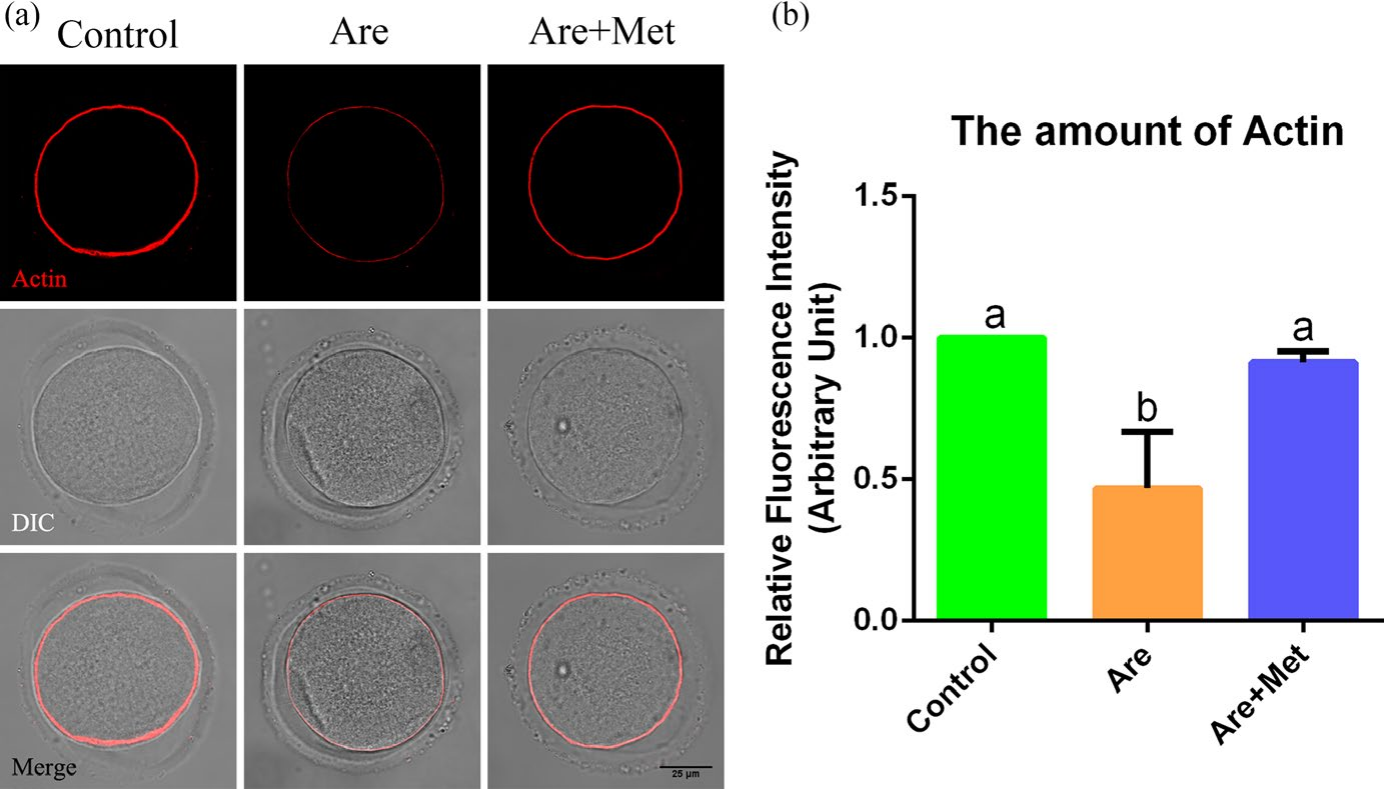
Effects of metformin on the actin dynamics in the arecoline‐exposed oocytes. A, Representative pictures of actin filaments (Red) in the control, Are and Are + Met groups. Scale bar = 25 μm. B, The relative fluorescence intensity of F‐actin in the control, Are and Are + Met groups. DIC, differential interference contrast. Data were presented as mean ± SD. ^a‐b^Means not sharing a common superscript are different (*P* < .05)

Actin filaments play a key role in cortical polarization and asymmetric spindle localization during oocyte maturation. To determine whether actin dynamics was involved in arecoline‐induced meiosis arrest, phalloidin‐TRITC was applied to stain the F‐actin. As shown in Figure 2A, actin filaments were evenly accumulated on the plasma membrane with robust fluorescent signal in the control oocytes. In contrast, the arecoline‐exposed oocytes displayed intermittent distribution of actin filaments with faded fluorescent signals, and these could be rescued to a level indistinguishable from the controls (Figure 2B; n = 222), implying that metformin prevented the actin dynamics from damage induced by arecoline.

### 3.4 Metformin rescued the spindle defects and chromosome misalignment in arecoline‐exposed oocytes

**Figure 3:**
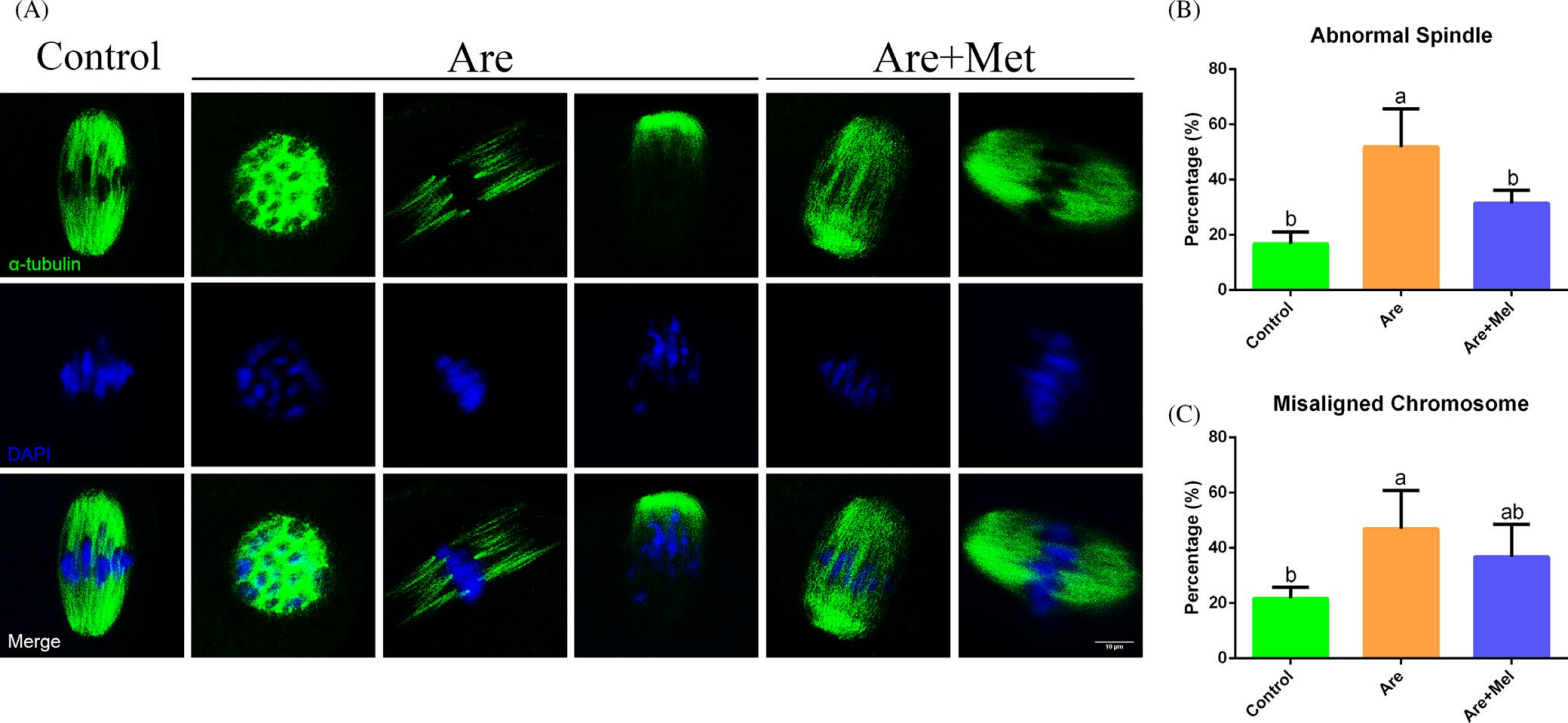
Effects of metformin on the spindle assembly and chromosome alignment in the arecoline‐exposed oocytes. A, Representative pictures of spindle morphologies and chromosome alignment in the control, Are and Are + Met groups. Spindle (green) and chromosome (blue). Scale bar = 10 μm. B, The rates of aberrant spindles in the control, Are and Are + Met groups. C, The rates of misaligned chromosomes in the control, Are and Are + Met groups. Data were presented as mean ± SD. ^a‐b^Means not sharing a common superscript are different (*P* < .05)

Due to meiotic failure of oocytes is highly correlated with defective spindle assembly and chromosome alignment, we next detected the spindle apparatus formation in metaphase I (MI) stage oocytes. About 8 hours after retrieval, oocytes in MI were immunostained with anti‐α‐tubulin‐FITC antibody to label the spindle organization and counterstained with DAPI to visualize the chromosome. The results showed that the majority of oocyte in control group exhibited a typical barrel‐shaped spindle with a well‐aligned chromosome on the equatorial plate (Figure 3A, left). However, a higher frequency of various morphology‐aberrant spindles with misaligned chromosomes was present in arecoline‐exposed oocytes (Figure 3A, middle). As expected, metformin treatment in arecoline‐exposed oocytes reduced partially the abnormal rates (Figure 3A, right and Figure 3B and C; n = 576; abnormal spindle: 17.36 ± 3.72%, n = 172 *vs.* 51.86 ± 13.77%, n = 268 *vs.* 31.47 ± 4.70%, n = 182, *P* < .01; misaligned chromosome: 22.03 ± 3.26%, n = 164 *vs.* 46.88 ± 13.90%, n = 176 *vs.* 36.77 ± 11.73%, n = 190, *P* < .05). All these results illustrated metformin could restore the dynamic stability of the oocyte cytoskeleton.

### 3.5 Metformin maintains euploidy by restoring defective kinetochore‐microtubule attachment in arecoline‐exposed oocytes

**Figure 4:**
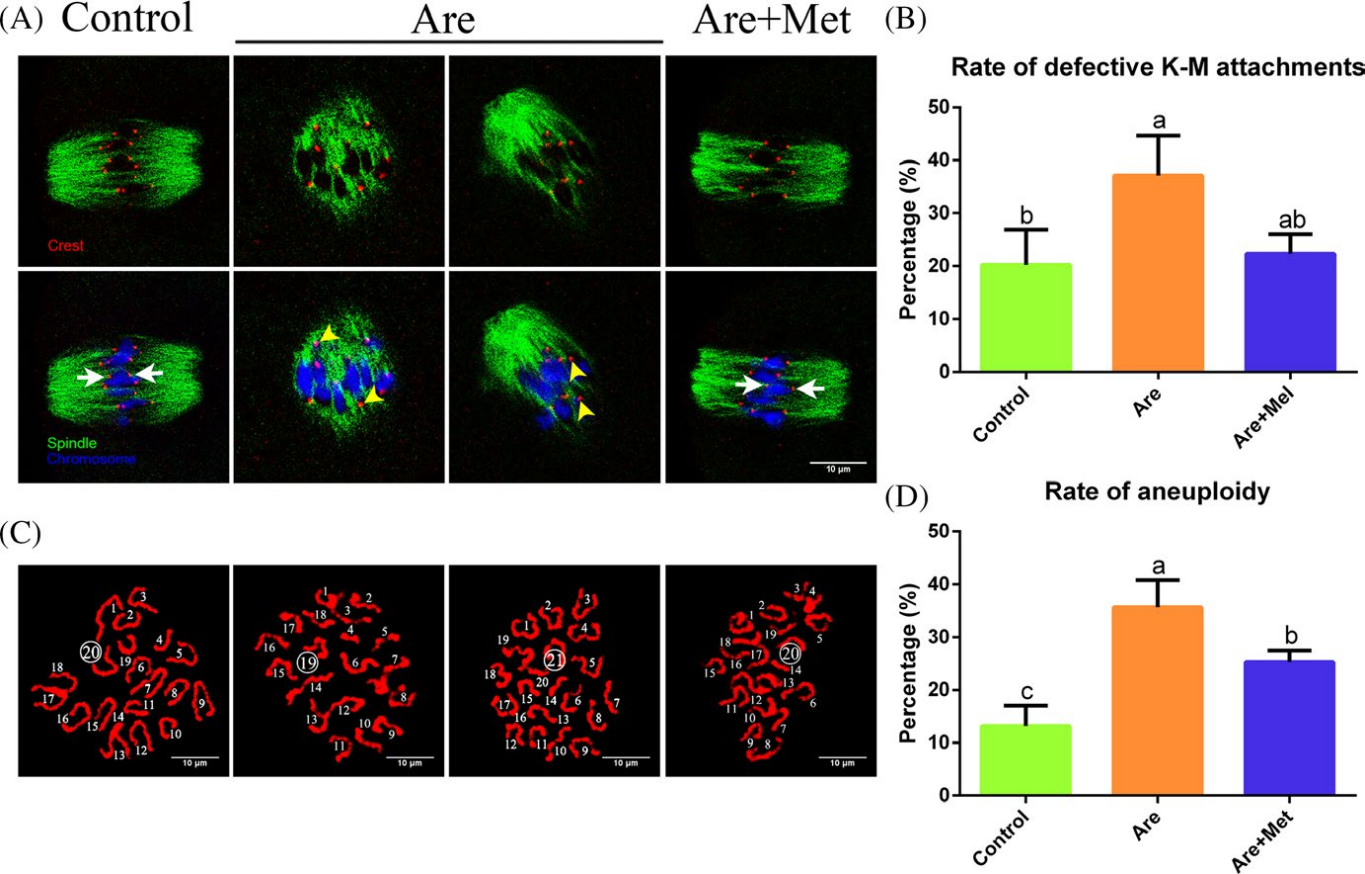
Effects of metformin on the kinetochore‐microtubule (K‐M) attachment and karyotype in the arecoline‐exposed oocytes. A, Typical images of K‐M attachment in the control, Are and Are + Met groups. Kinetochore (red), microtubule (green) and chromosome (blue). The white arrow represents normal attachments; the yellow arrowhead represents abnormal attachments. B, The rates of defective K‐M attachments in the control, Are and Are + Met groups. C, Typical images of euploidy (20 univalents) and aneuploidy (less or more than 20 univalents). D, The rates of aneuploid oocytes in the control, Are and Are + Met groups. Scale bar = 10 μm. Data were presented as mean ± SD. ^a‐c^Means not sharing a common superscript are different (*P* < .05)

Abnormal spindle formation and chromosome alignment are often associated with the aneuploidy which is usually caused by the defective K‐M attachment. Thus, we tested the stability of K‐M attachments. As shown in Figure 4A, the kinetochore and microtubule were completely attached in control group. The arecoline‐exposed oocytes showed defective K‐M attachment. However, the rate of aberrant K‐M attachments caused by arecoline in metformin‐rescued group reduced to an indistinguishable level compared with the control (Figure 4A and B; 20.21 ± 6.68%, n = 156 *vs.* 37.10 ± 7.60%, n = 150 *vs.* 22.35 ± 3.67%, n = 164, *P* < .05).

In addition, we analysed the karyotype of MII eggs by chromosome spread assay. Aneuploidy is defined as less than or more than 20 univalents. The results indicated that aneuploidy rate obviously raised in arecoline‐exposed oocytes compared to control, with statistical differences, whereas metformin partially reduced the rate of aneuploidy induced by arecoline (Figure 4C and D; 13.18 ± 3.83%, n = 134 *vs.* 35.68 ± 5.14%, n = 164 *vs.* 25.31 ± 2.15%, n = 148, *P* < .01). All these data suggested that arecoline exposure enhanced aneuploidy by inducing cytoskeletal defects, ultimately leading to oocyte meiosis failure, and metformin treatment could break this arrest and restore the meiotic progression of mouse oocytes.

### 3.6 Metformin elevates the rate of homogeneous mitochondrial distribution, ATP content and mitochondrial membrane potential in arecoline‐exposed oocytes

**Figure 5:**
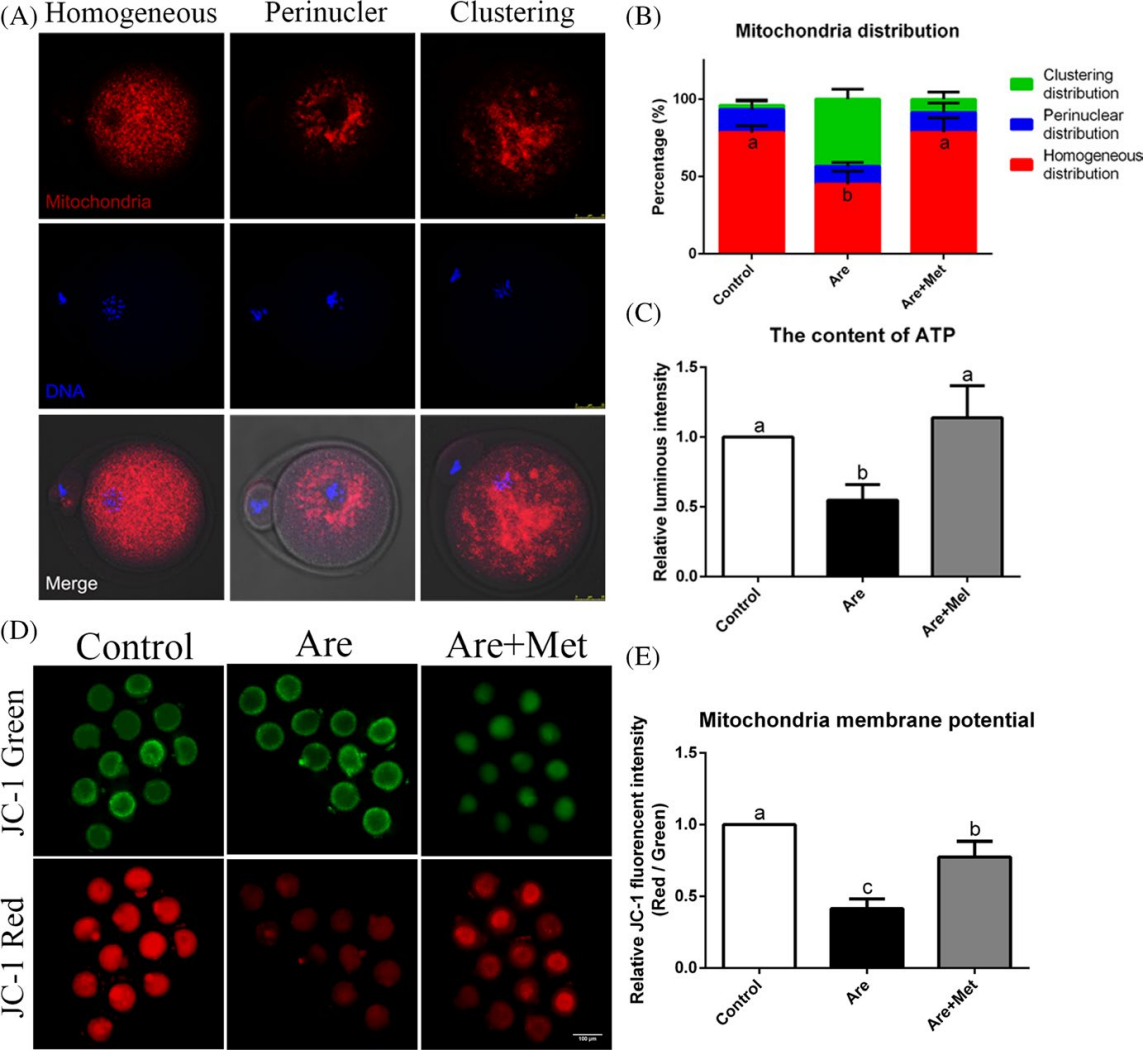
Effects of metformin on mitochondrial distribution, ATP content and mitochondrial membrane potential in the arecoline‐exposed oocytes. A, Representative photographs of homogenous, perinuclear and clustering distribution of mitochondria in mouse oocytes. Mitochondria (red) and chromosome (blue) were shown. Scale bar = 25 μm. B, The rates of homogeneous, perinuclear and clustering mitochondrial distribution pattern. C, The content of ATP in the control, Are and Are + Met groups. D, Mitochondrial membrane potential was calculated as the ratio of red florescence, which corresponds to activated mitochondria (J‐aggregates), to green fluorescence, which corresponds to less‐activated mitochondria (J‐monomers). Scale bar = 100 μm. E, Relative fluorescence intensity of the mitochondrial membrane potential in the control, Are and Are + Met groups. Data were presented as mean ± SD. ^a‐b^Means not sharing a common superscript are different (*P* < .05)

The functional integrity of mitochondria is critical for oocyte meiosis and considered to be one of the key indicators of oocyte cytoplasmic maturation. We then detected the mitochondrial distribution of MII stage eggs to research whether arecoline exposure would result in mitochondrial dysfunction. As shown in Figure 5A, three typical images from left to right represented homogenous, perinuclear and cluster distribution of mitochondria, respectively. Compared with the control oocytes, arecoline notably reduced the rate of homogenous distribution, which could be restored in metformin‐rescued oocyte (Figure 5B; homogenous distribution: 78.33 ± 4.41%, n = 186 *vs.* 45.06 ± 8.37%, n = 186 *vs.* 78.38 ± 9.14%, n = 192, *P* < .01).

ATP content is closely related to mitochondrial function. Next, we examined the ATP content of oocytes in different treatment groups. Not surprisingly, arecoline did significantly decreased the ATP content of oocytes compared to the control, while metformin returned ATP to normal levels after supplementation (Figure 5C; n = 405). These results showed metformin could increase ATP production by repairing mitochondrial dysfunction in arecoline‐exposed oocytes.

To exploring the mechanism of ATP reduction, mitochondrial membrane potential in each group was detected. The results revealed that mitochondrial Δψm sharply decreased after arecoline treatment (Figure 5D and E; n = 375). In addition, the reduction of Δψm could be present in the presence of metformin.

### 3.7 Metformin reduces ROS level and limits early apoptosis in arecoline‐exposed oocytes

**Figure 6:**
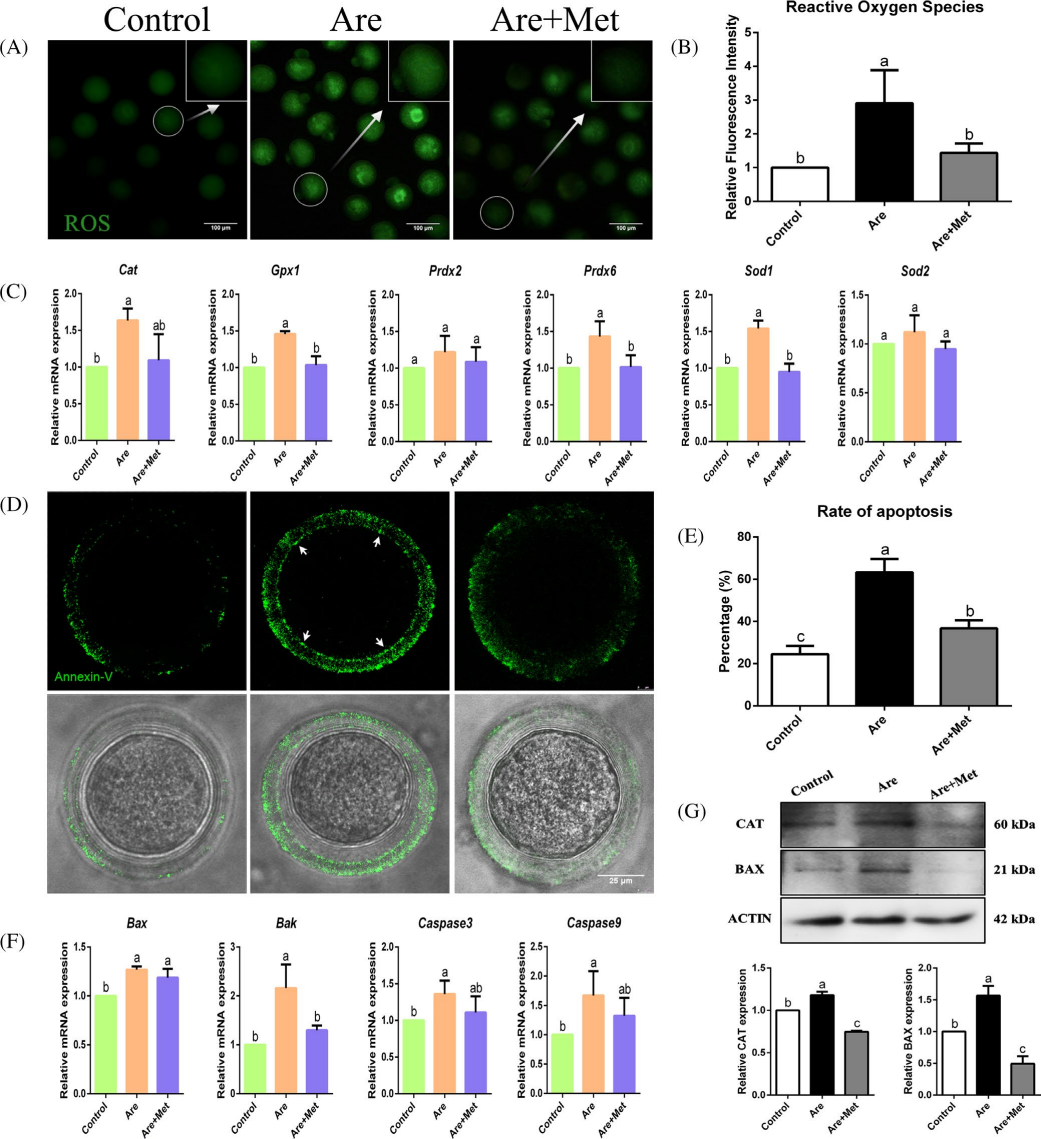
Effects of metformin on oxidative stress and early apoptosis in the arecoline‐exposed oocytes. A, Representative photographs of ROS levels (green) in the control, Are and Are + Met groups. Scale bar = 100 μm. B, Relative fluorescence intensity of ROS in the control, Are and Are + Met groups. C, Relative expression of oxidative stress‐related genes, including *Cat*, *Gpx1*, *Prdx2*, *Prdx6*, *Sod2* and *Sod1*. D, Representative photographs of early apoptotic oocytes in the control, Are and Are + Met groups. Oocytes with fluorescence signals (green) at both membrane (white arrow) and zona pellucida are regarded as early‐stage apoptosis. Scale bar = 25 μm. E, The rate of early apoptosis oocytes in the control, Are and Are + Met groups. F, Relative expression of apoptosis‐related genes, including *Bax*, *Bak*, *Caspase3* and *Caspase9*. (g) Relative level of oxidative stress and apoptosis‐related proteins, including CAT and BAX. Data were presented as mean ± SD. ^a‐c^Means not sharing a common superscript are different (*P* < .05)

Furthermore, oxidative stress is usually induced by mitochondrial dysfunction, which speeds up the progression of apoptosis. Thus, we investigated the extent of ROS generation in arecoline‐exposed and metformin‐rescued oocytes. As shown in Figure 6A, the oxidative stress signal of the arecoline‐exposed oocytes was significantly increased compared with the control, while obviously reduced in metformin‐rescued oocytes. Fluorescence intensity analysis was also consistent with the above observations (Figure 6B; n = 574), which was further confirmed by qRT‐PCR (Figure 6C; n = 520) and Western blot (Figure 6G, upper panel; n = 453).

Cumulative studies have shown that excessive ROS could induce oocyte apoptosis, and we next explored the early apoptosis of oocytes. It is worth noting that only oocytes with green fluorescent signals in both the zona pellucida and the cytoplasmic membrane are considered to have experienced early apoptosis.^48^ Our results showed that the number of oocytes undergone early apoptosis in the arecoline‐exposed group was raised dramatically, whereas the metformin‐rescued group was reduced to a level indistinguishable from the control group (Figure 6D and E; 24.51 ± 3.88%, n = 201 *vs* 63.28 ± 6.34%, n = 207 *vs* 36.75 ± 3.84%, n = 186, *P* < .01). In addition, the results of qPCR (Figure 6F; n = 330) and immunoblotting (Figure 6G, lower panel; n = 369) showed apoptosis‐related genes and protein expression levels, which further verified the above results. The data suggested that arecoline induced a decrease in ATP production and increased ROS synthesis by disrupting mitochondrial integrity, ultimately leading to oocyte apoptosis. Surprisingly, metformin administration partially relieved these damages caused by arecoline exposure.

## 4 DISCUSSION

In the present study, we found arecoline displayed extremely toxicity effect on mammalian oocytes maturation via regulating ROS level, further injuring mitochondrial function. Many studies revealed that metformin could reduce ROS content in several kinds of cells, and display positive effects in oocytes maturation and further embryonic development.^43^, ^44^, ^45^ According to the information, we hypothesized that metformin could rescue arecoline‐induced oocyte cytotoxicity.

To confirm our hypothesis, we first examined the effect of arecoline on GVBD and the PBE of mouse oocytes, which is a sign of meiotic resumption^49^ and nuclear maturation,^50^ respectively. Oocyte maturation is the key event of life initial. Poor quality of matured oocytes directly results in failure of embryo development, lower pregnancy rate and abnormal offspring.^51^ Oocyte maturation includes nuclear maturation, which marked as the 1st polar body extrusion, and cytoplasm maturation, which marked as mono‐spermic penetration, nuclear reprogramming and embryonic development.^52^

Nuclear maturation is primarily involved in chromosome segregation, which is easily detected by the PBE. Therefore, the PBE failure indicates abnormal chromosome separation, which is usually caused by a damaged cytoskeleton. In oocytes, actin filaments and microtubules are the most important components of the cytoskeleton and play a key role in ensuring proper chromosome segregation during meiosis.^53^ Studies have shown that abnormal spindle assembly and chromosomal misalignment can impair female fertility in different species.^54^ Our data indicated that arecoline disrupts actin filament dynamics and spindle assembly, suggesting that arecoline destroys cytoskeletal integrity. Compared to the control, metformin partially or completely restored the actin dynamics, spindle defects and chromosomal misalignment in the arecoline‐exposed oocytes to indistinguishable levels. Thus, metformin safeguards the cytoskeletal integrity of the arecoline‐exposed oocytes and ensures nuclear maturation during oocyte meiosis.

Abnormal cytoskeleton, especially spindle defects and chromosomal misalignment, is always accompanied by errors in K‐M attachment, which usually results in oocyte aneuploidy.^55^ Therefore, we examined the K‐M attachment stability and euploidy in arecoline‐exposed oocytes. The results indicate that arecoline perturbs the stability of K‐M attachment and obviously enhances the aneuploidy of oocytes, but these could be rescued by metformin treatment. Therefore, arecoline exposure enhanced aneuploidy by inducing cytoskeletal defects, ultimately leading to oocyte meiosis failure, and metformin treatment could break this arrest and restore the meiotic progression of mouse oocytes.

Arecoline treatment caused widely abnormal on oocyte maturation in the present study, which revealed that arecoline disturbed some basic cell function, further results in the abnormal of nuclear and cytoplasmic dynamics. Almost all cell event associated with energy supply. Metabolic and oxidation respiration are the basic characteristics of life. According to this, we doubt that arecoline inhibit the function of mitochondrial, further induced abnormal of maturation.

Mitochondria are important organelles to produce ATP in oocytes, and their functional integrity is critical for oocyte meiosis and is considered as a key indicators of oocyte cytoplasmic maturation. Mitochondria disperse from the perinuclear area to the cytoplasm during meiotic maturation of oocytes, and their failure to migrate usually marks a failure of cytoplasmic maturation. So next, we examined the distribution of mitochondria in the arecoline‐exposed oocytes and then tested the ATP content. The results confirmed that metformin could increase ATP production by repairing mitochondrial dysfunction in the arecoline‐exposed oocytes. In addition, studies have found that excessive oxidative stress can impede cytoplasmic maturation and embryonic development of oocytes.^56^, ^57^ At the same time, ROS accumulation leads to mitochondrial dysfunction and induces oocyte apoptosis. Our data confirmed that arecoline induces a large amount of ROS, resulting in significant apoptosis of oocytes. And these could be restored after metformin supplementation. These results reveal that arecoline induces a decrease in ATP production and an increase in ROS synthesis by disrupting mitochondrial integrity, ultimately leading to oocyte apoptosis. Fortunately, metformin administration partially alleviates these damages caused by arecoline exposure.

To further explore the specific mechanism of apoptosis caused by arecoline, we examined the mitochondrial membrane potential of oocytes, and the results showed that arecoline significantly reduced the membrane potential of mitochondria. Our previous studies have shown that the decrease of mitochondrial membrane potential in oocytes can cause the release of cytochrome *c*, which in turn activates the up‐regulation of intracellular apoptosis‐related caspase and leads to apoptosis of oocytes.^27^, ^58^, ^59^ To establish whether this same apoptotic mechanism also occurs in arecoline‐exposed oocytes, the expression levels of oxidative stress and apoptosis‐related genes and proteins further were validated. Our results clearly revealed that caspase signalling pathway–related genes and proteins *Bax*, *Bak*, *Caspase9* and *Caspase3* were significantly elevated compared with the control group.

Embryonic development which is related with complex biological process includes genome reprogramming^60^ and zygote genomic activation.^61^ Although metformin successfully rescued the nuclear maturation of mouse oocytes in the presence of arecoline, the embryo development after parthenote activation is still very poor. The cause for this result may be that the cytoplasmic damage caused by arecoline to oocytes did not been completely recovered by metformin, that is, the cytoplasm did not fully matured. The results indicated that arecoline treatment caused seriously and widely damage on oocyte. However, the deep molecular mechanism should be explored in the future.

Mechanism of metformin on ROS content and oocyte quality regulation has been well explored. Previous studies reported that metformin inhibits the activity of complex I (NADH) of the mitochondrial respiratory chain,^62^, ^63^, ^64^, ^65^ further reduced production of ROS. This inhibition next led to activation of the AMP‐activated protein kinase (AMPK) complex.^66^ AMPK was a key cellular energy sensor to maintain the cellular energy homeostasis. Following AMPK activation, the catabolism pathway for energy production was initiated, including stimulating cellular glucose uptake and mitochondrial biosynthesis, and facilitating glycolysis, β‐oxidation of fatty acids and oxidative phosphorylation.^67^ This compensation mechanism was designed to restore sufficient energy to maintain cellular homeostasis.^68^, ^69^, ^70^

During maturation, oocytes obtain most of their energy from cumulus cells, which metabolize glucose through glycolysis to produce lactate and pyruvate, the preferred substrates for oocytes.^71^ Metformin has been reported to stimulate human granulosa cells to produce lactic acid,^72^ which can improve oocyte development and increase metabolic substrates for ATP production. Moreover, lipid was also an important energy substance for oocytes. Metformin can increase the usage of lipids and β‐oxidation of fatty acids, which was essential for mouse oocyte maturation and early embryo development.^34^, ^73^

**Figure 7:**
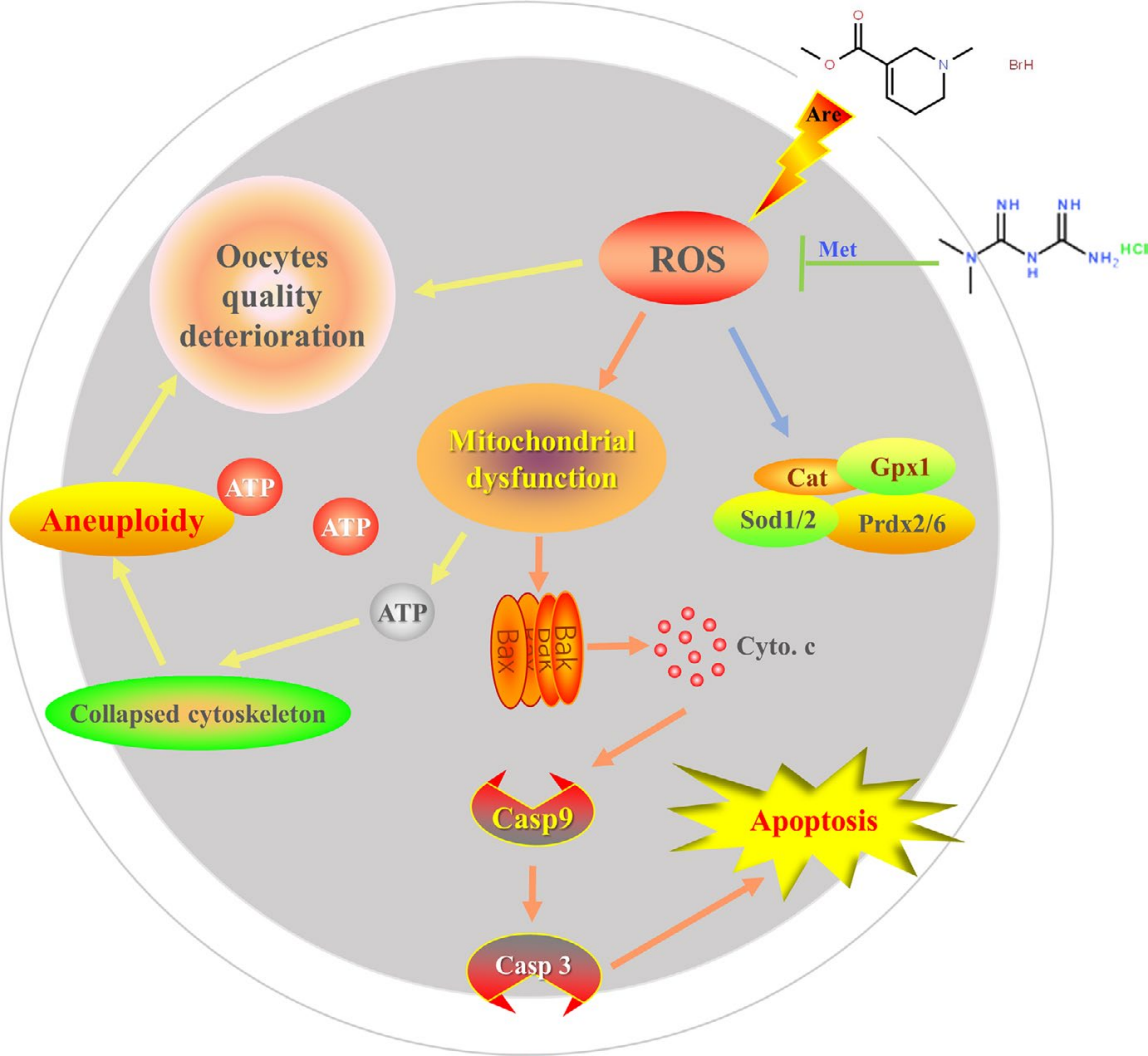
Potential mechanism of metformin‐mediated improvement of mouse oocyte apoptosis defects induced by arecoline. The arecoline leads to mitochondrial dysfunction by reducing the mitochondrial membrane potential of oocytes. On the one hand, it will cause a decrease in the production of ATP to reduce the energy supply, leading to the collapse of the oocyte cytoskeleton, which in turn causes aneuploidy, resulting in a deterioration in oocyte quality. On the other hand, a decrease in mitochondrial membrane potential causes the release of cytochrome *c*, which activates the caspase signalling pathway and ultimately leads to oocyte apoptosis. However, metformin could partially reduce these damages, suggesting that metformin has a protective effect against the apoptosis of oocytes induced by arecoline

In conclusion, as shown in Figure 7, our study demonstrates that arecoline leads to mitochondrial dysfunction by reducing the mitochondrial membrane potential of oocytes. Furthermore, the dysfunction of mitochondria decreases the production of ATP, leading to the collapse of the oocyte cytoskeleton, which in turn causes aneuploidy, resulting in a deterioration of oocyte quality. On the other hand, damnification of mitochondria membrane causes the release of cytochrome *c*, which activates the caspase signalling pathway and ultimately leads to oocyte apoptosis.^74^ Fortunately, metformin could partially reduce these damages, suggesting that metformin has a protective effect against the apoptosis of oocytes induced by arecoline.

## CONFLICT OF INTEREST

There is no conflict of interest in the declaration.

## AUTHORS’ CONTRIBUTIONS

WL, MZ and QS provided key intellectual input in the conception and design of these studies. WL and MZ wrote the manuscript. WL and CZ performed all experiments. SY and WS contributed to the writing of the manuscript. All authors reviewed the manuscript.

## Supporting information

Figure S1

## Data Availability

The data used to support the findings of this study are available from the corresponding author upon request.

## References

[1] Gupta PC , Warnakulasuriya S . Global epidemiology of areca nut usage. Addict Biol. 2002;7(1):77‐83. 11900626 10.1080/13556210020091437

[2] Garg A , Chaturvedi P , Gupta PC . A review of the systemic adverse effects of areca nut or betel nut. Indian J Med Paediatr Oncol. 2014;35(1):3‐9. 25006276 10.4103/0971-5851.133702 PMC4080659

[3] Winstock AR , Trivedy CR , Warnakulasuriya KA , Peters TJ . A dependency syndrome related to areca nut use: some medical and psychological aspects among areca nut users in the Gujarat community in the UK. Addict Biol. 2000;5(2):173‐179. 20575832 10.1080/13556210050003766

[4] Boucher BJ , Mannan N . Metabolic effects of the consumption of Areca catechu. Addict Biol. 2002;7(1):103‐110. 11900629 10.1080/13556210120091464

[5] Gilani AH , Ghayur MN , Saify ZS , Ahmed SP , Choudhary MI , Khalid A . Presence of cholinomimetic and acetylcholinesterase inhibitory constituents in betel nut. Life Sci. 2004;75(20):2377‐2389. 15350815 10.1016/j.lfs.2004.03.035

[6] Lim DY , Kim IS . Arecoline inhibits catecholamine release from perfused rat adrenal gland. Acta Pharmacol Sin. 2006;27(1):71‐79. 16364213 10.1111/j.1745-7254.2006.00233.x

[7] Humans IWGotEoCRt . Betel‐quid and areca‐nut chewing and some areca‐nut derived nitrosamines. IARC Monogr Eval Carcinog Risks Hum. 2004;85:1‐334. 15635762 PMC4781453

[8] Cai ZY , Li YC , Li LH , Chen ZG . Analysis of arecoline in Semen Arecae decoction pieces by microchip capillary electrophoresis with contactless conductivity detection. J Pharm Anal. 2012;2(5):356‐360. 29403766 10.1016/j.jpha.2012.07.003 PMC5760757

[9] Chang YF , Liu TY , Liu ST , Tseng CN . Arecoline inhibits myogenic differentiation of C2C12 myoblasts by reducing STAT3 phosphorylation. Food Chem Toxicol. 2012;50(10):3433‐3439. 22847137 10.1016/j.fct.2012.07.032

[10] Tseng SK , Chang MC , Su CY et al Arecoline induced cell cycle arrest, apoptosis, and cytotoxicity to human endothelial cells. Clin Oral Invest. 2012;16(4):1267‐1273. 10.1007/s00784-011-0604-1 21847594

[11] Shih YT , Chen PS , Wu CH , Tseng YT , Wu YC , Lo YC . Arecoline, a major alkaloid of the areca nut, causes neurotoxicity through enhancement of oxidative stress and suppression of the antioxidant protective system. Free Radic Biol Med. 2010;49(10):1471‐1479. 20691257 10.1016/j.freeradbiomed.2010.07.017

[12] Chatterjee A , Deb S . Genotoxic effect of arecoline given either by the peritoneal or oral route in murine bone marrow cells and the influence of N‐acetylcysteine. Cancer Lett. 1999;139(1):23‐31. 10408905 10.1016/s0304-3835(98)00364-4

[13] Chang LY , Lai YL , Yu TH , Chen YT , Hung SL . Effects of areca nut extract on lipopolysaccharides‐enhanced adhesion and migration of human mononuclear leukocytes. J Periodontol. 2014;85(6):859‐867. 24001044 10.1902/jop.2013.130198

[14] Er TK , Tsai EM , Tsai LY , Ko YC , Lee JN . In vitro effects of arecoline on sperm motility and cyclooxygenase‐2 expression. J Toxicol Sci. 2006;31(1):75‐82. 16538046 10.2131/jts.31.75

[15] Wu PF , Chiang TA , Chen MT et al A characterization of the antioxidant enzyme activity and reproductive toxicity in male rats following sub‐chronic exposure to areca nut extracts. J Hazard Mater. 2010;178(1–3):541‐546. 20202746 10.1016/j.jhazmat.2010.01.118

[16] Yang MS , Lee CH , Chang SJ et al The effect of maternal betel quid exposure during pregnancy on adverse birth outcomes among aborigines in Taiwan. Drug Alcohol Depend. 2008;95(1–2):134‐139. 18282667 10.1016/j.drugalcdep.2008.01.003

[17] Senn M , Baiwog F , Winmai J , Mueller I , Rogerson S , Senn N . Betel nut chewing during pregnancy, Madang province, Papua New Guinea. Drug Alcohol Depend. 2009;105(1–2):126‐131. 19665325 10.1016/j.drugalcdep.2009.06.021

[18] Chue AL , Carrara VI , Paw MK et al Is areca innocent? The effect of areca (betel) nut chewing in a population of pregnant women on the Thai‐Myanmar border. Int Health. 2012;4(3):204‐209. 24029401 10.1016/j.inhe.2012.05.001 PMC3442179

[19] Chang BE , Liao MH , Kuo MY , Chen CH . Developmental toxicity of arecoline, the major alkaloid in betel nuts, in zebrafish embryos. Birth Defects Res A Clin Mol Teratol. 2004;70(1):28‐36. 14745892 10.1002/bdra.10136

[20] Peng WH , Lee YC , Chau YP , Lu KS , Kung HN . Short‐term exposure of zebrafish embryos to arecoline leads to retarded growth, motor impairment, and somite muscle fiber changes. Zebrafish. 2015;12(1):58‐70. 25549301 10.1089/zeb.2014.1010 PMC4298148

[21] Liu ST , Young GC , Lee YC , Chang YF . A preliminary report on the toxicity of arecoline on early pregnancy in mice. Food Chem Toxicol. 2011;49(1):144‐148. 20940028 10.1016/j.fct.2010.10.009

[22] Li WD , Yu S , Luo SM , Shen W , Yin S , Sun QY . Melatonin defends mouse oocyte quality from benzo[ghi]perylene‐induced deterioration. J Cell Physiol. 2019;234(5):6220‐6229. 30317565 10.1002/jcp.27351

[23] Miao YL , Zhang X , Zhao JG et al Effects of griseofulvin on in vitro porcine oocyte maturation and embryo development. Environ Mol Mutagen. 2012;53(7):561‐566. 22829310 10.1002/em.21717

[24] Kim SH , Zhao MH , Liang S , Cui XS , Kim NH . Inhibition of cathepsin B activity reduces apoptosis by preventing cytochrome c release from mitochondria in porcine parthenotes. J Reprod Dev. 2015;61(4):261‐268. 25903788 10.1262/jrd.2015-019 PMC4547983

[25] Hu DB , Li ZS , Ali I , Xu LJ , Fang NZ . Effect of potential role of p53 on embryo development arrest induced by H2O2 in mouse. Vitro Cell Dev Biol Anim. 2017;53(4):344‐353. 10.1007/s11626-016-0122-1 28127704

[26] Leist M , Nicotera P . The shape of cell death. Biochem Biophys Res Commun. 1997;236(1):1‐9. 9223415 10.1006/bbrc.1997.6890

[27] Zhao MH , Kim NH , Cui XS . GlutaMAX prolongs the shelf life of the culture medium for porcine parthenotes. Theriogenology. 2016;85(3):368‐375. 26462658 10.1016/j.theriogenology.2015.08.014

[28] Inzucchi SE , Bergenstal RM , Buse JB et al Management of hyperglycemia in type 2 diabetes: a patient‐centered approach: position statement of the American Diabetes Association (ADA) and the European Association for the Study of Diabetes (EASD). Diabetes Care. 2012;35(6):1364‐1379. 22517736 10.2337/dc12-0413 PMC3357214

[29] Evans JM , Donnelly LA , Emslie‐Smith AM , Alessi DR , Morris AD . Metformin and reduced risk of cancer in diabetic patients. BMJ. 2005;330(7503):1304‐1305. 15849206 10.1136/bmj.38415.708634.F7 PMC558205

[30] Franciosi M , Lucisano G , Lapice E , Strippoli GF , Pellegrini F , Nicolucci A . Metformin therapy and risk of cancer in patients with type 2 diabetes: systematic review. PLoS ONE. 2013;8(8):e71583. 23936520 10.1371/journal.pone.0071583 PMC3732236

[31] Gui DY , Sullivan LB , Luengo A et al Environment dictates dependence on mitochondrial complex I for NAD+ and aspartate production and determines cancer cell sensitivity to metformin. Cell Metab. 2016;24(5):716‐727. 27746050 10.1016/j.cmet.2016.09.006 PMC5102768

[32] Wu D , Hu D , Chen H et al Glucose‐regulated phosphorylation of TET2 by AMPK reveals a pathway linking diabetes to cancer. Nature. 2018;559(7715):637‐641. 30022161 10.1038/s41586-018-0350-5 PMC6430198

[33] Cabreiro F , Au C , Leung KY et al Metformin retards aging in C. elegans by altering microbial folate and methionine metabolism. Cell. 2013;153(1):228‐239. 23540700 10.1016/j.cell.2013.02.035 PMC3898468

[34] Martin‐Montalvo A , Mercken EM , Mitchell SJ et al Metformin improves healthspan and lifespan in mice. Nat Commun. 2013;4:2192. 23900241 10.1038/ncomms3192 PMC3736576

[35] Barzilai N , Crandall JP , Kritchevsky SB , Espeland MA . Metformin as a Tool to Target Aging. Cell Metab. 2016;23(6):1060‐1065. 27304507 10.1016/j.cmet.2016.05.011 PMC5943638

[36] Fang J , Yang J , Wu X et al Metformin alleviates human cellular aging by upregulating the endoplasmic reticulum glutathione peroxidase 7. Aging Cell. 2018;17(4):e12765. 29659168 10.1111/acel.12765 PMC6052468

[37] Blagih J , Coulombe F , Vincent EE et al The energy sensor AMPK regulates T cell metabolic adaptation and effector responses in vivo. Immunity. 2015;42(1):41‐54. 25607458 10.1016/j.immuni.2014.12.030

[38] Eikawa S , Nishida M , Mizukami S , Yamazaki C , Nakayama E , Udono H . Immune‐mediated antitumor effect by type 2 diabetes drug, metformin. Proc Natl Acad Sci USA. 2015;112(6):1809‐1814. 25624476 10.1073/pnas.1417636112 PMC4330733

[39] Ursini F , Russo E , Pellino G et al Metformin and autoimmunity: A "New Deal" of an old drug. Front Immunol. 2018;9 10.3389/fimmu.2018.01236 PMC5994909 29915588

[40] Palomba S , Orio F Jr , Falbo A , Russo T , Tolino A , Zullo F . Clomiphene citrate versus metformin as first‐line approach for the treatment of anovulation in infertile patients with polycystic ovary syndrome. J Clin Endocrinol Metab. 2007;92(9):3498‐3503. 17595241 10.1210/jc.2007-1009

[41] Palomba S , Falbo A , Russo T , Orio F , Tolino A , Zullo F . Systemic and local effects of metformin administration in patients with polycystic ovary syndrome (PCOS): relationship to the ovulatory response. Hum Reprod. 2010;25(4):1005‐1013. 20106839 10.1093/humrep/dep466

[42] Sabatini ME , Guo L , Lynch MP et al Metformin therapy in a hyperandrogenic anovulatory mutant murine model with polycystic ovarian syndrome characteristics improves oocyte maturity during superovulation. J Ovarian Res. 2011;4(1):8. 21605417 10.1186/1757-2215-4-8 PMC3121715

[43] Ghasemnejad‐Berenji M , Ghazi‐Khansari M , Yazdani I et al Effect of metformin on germ cell‐specific apoptosis, oxidative stress and epididymal sperm quality after testicular torsion/detorsion in rats. Andrologia. 2018;50(2):e12846. 10.1111/and.12846 28730645

[44] Huang Y , Yu Y , Gao J et al Impaired oocyte quality induced by dehydroepiandrosterone is partially rescued by metformin treatment. PLoS ONE. 2015;10(3):e0122370. 25811995 10.1371/journal.pone.0122370 PMC4374838

[45] Louden ED , Luzzo KM , Jimenez PT , Chi T , Chi M , Moley KH . TallyHO obese female mice experience poor reproductive outcomes and abnormal blastocyst metabolism that is reversed by metformin. Reprod Fertil Dev. 2014;27(1):31‐39. 25472042 10.1071/RD14339 PMC4365903

[46] Sun QY , Wu GM , Lai L et al Translocation of active mitochondria during pig oocyte maturation, fertilization and early embryo development in vitro. Reproduction. 2001;122(1):155‐163. 11425340

[47] Ge H , Tollner TL , Hu Z et al The importance of mitochondrial metabolic activity and mitochondrial DNA replication during oocyte maturation in vitro on oocyte quality and subsequent embryo developmental competence. Mol Reprod Dev. 2012;79(6):392‐401. 22467220 10.1002/mrd.22042

[48] Duan X , Wang QC , Chen KL , Zhu CC , Liu J , Sun SC . Acrylamide toxic effects on mouse oocyte quality and fertility in vivo. Sci Rep. 2015;5:11562. 26108138 10.1038/srep11562 PMC4479821

[49] Sun QY , Miao YL , Schatten H . Towards a new understanding on the regulation of mammalian oocyte meiosis resumption. Cell Cycle. 2009;8(17):2741‐2747. 19717979 10.4161/cc.8.17.9471

[50] Funahashi H , Day BN . Effects of the duration of exposure to hormone supplements on cytoplasmic maturation of pig oocytes in vitro. J Reprod Fertil. 1993;98(1):179‐185. 8345463 10.1530/jrf.0.0980179

[51] Marteil G , Richard‐Parpaillon L , Kubiak JZ . Role of oocyte quality in meiotic maturation and embryonic development. Reprod Biol. 2009;9(3):203‐224. 19997475 10.1016/s1642-431x(12)60027-8

[52] Eppig JJ . Coordination of nuclear and cytoplasmic oocyte maturation in eutherian mammals. Reprod Fertil Dev. 1996;8(4):485‐489. 8870074 10.1071/rd9960485

[53] Sanfins A , Lee GY , Plancha CE , Overstrom EW , Albertini DF . Distinctions in meiotic spindle structure and assembly during in vitro and in vivo maturation of mouse oocytes. Biol Reprod. 2003;69(6):2059‐2067. 12930715 10.1095/biolreprod.103.020537

[54] Herrick JR , Brad AM , Krisher RL . Chemical manipulation of glucose metabolism in porcine oocytes: effects on nuclear and cytoplasmic maturation in vitro. Reproduction. 2006;131(2):289‐298. 16452722 10.1530/rep.1.00835

[55] Shomper M , Lappa C , FitzHarris G . Kinetochore microtubule establishment is defective in oocytes from aged mice. Cell Cycle. 2014;13(7):1171‐1179. 24553117 10.4161/cc.28046 PMC4013167

[56] Dennery PA . Effects of oxidative stress on embryonic development. Birth Defects Res C Embryo Today. 2007;81(3):155‐162. 17963268 10.1002/bdrc.20098

[57] Lange G , Mohr P . Pharyngeal diphtheria diagnosed too late, fatal course of the disease. HNO. 1988;36(12):498‐501. 3235363

[58] Kaulen DR , Golovanova TA , Pyatykhina DP , Khorobrykh VV . Inhibition of hematopoietic stem cells by syngeneic lymphocytes. Bull Exp Biol Med. 1974;77(2):156‐158. 4547983 10.1007/BF00809620

[59] Shekhtman MM . Mitral commissurotomy and pregnancy. Med Sestra. 1974;33(12):28‐29. 4498905

[60] Langhorst ML . Monitoring airborne reactive chemicals by derivatization and high performance thin layer chromatography–anhydrides, acid halides, isocyanates. Am Ind Hyg Assoc J. 1985;46(5):236‐243. 4003274 10.1080/15298668591394743

[61] Latham KE , Schultz RM . Embryonic genome activation. Front Biosci. 2001;6:D748‐759. 11401780 10.2741/latham

[62] El‐Mir MY , Nogueira V , Fontaine E , Averet N , Rigoulet M , Leverve X . Dimethylbiguanide inhibits cell respiration via an indirect effect targeted on the respiratory chain complex I. J Biol Chem. 2000;275(1):223‐228. 10617608 10.1074/jbc.275.1.223

[63] Owen MR , Doran E , Halestrap AP . Evidence that metformin exerts its anti‐diabetic effects through inhibition of complex 1 of the mitochondrial respiratory chain. Biochem J. 2000;348(Pt 3):607‐614. 10839993 PMC1221104

[64] Cameron AR , Logie L , Patel K et al Metformin selectively targets redox control of complex I energy transduction. Redox Biol. 2018;14:187‐197. 28942196 10.1016/j.redox.2017.08.018 PMC5609876

[65] Detaille D , Guigas B , Leverve X , Wiernsperger N , Devos P . Obligatory role of membrane events in the regulatory effect of metformin on the respiratory chain function. Biochem Pharmacol. 2002;63(7):1259‐1272. 11960602 10.1016/s0006-2952(02)00858-4

[66] Hardie DG , Ross FA , Hawley SA . AMPK: a nutrient and energy sensor that maintains energy homeostasis. Nat Rev Mol Cell Biol. 2012;13(4):251‐262. 22436748 10.1038/nrm3311 PMC5726489

[67] Faure M , Bertoldo MJ , Khoueiry R et al Metformin in Reproductive Biology. Front Endocrinol. 2018;9:675. 10.3389/fendo.2018.00675 PMC6262031 30524372

[68] Ke R , Xu Q , Li C , Luo L , Huang D . Mechanisms of AMPK in the maintenance of ATP balance during energy metabolism. Cell Biol Int. 2018;42(4):384‐392. 29205673 10.1002/cbin.10915

[69] Jager S , Handschin C , St‐Pierre J , Spiegelman BM . AMP‐activated protein kinase (AMPK) action in skeletal muscle via direct phosphorylation of PGC‐1. Proc Natl Acad Sci. 2007;104(29):12017‐12022 17609368 10.1073/pnas.0705070104 PMC1924552

[70] Nascimben L , Ingwall JS , Lorell BH et al Mechanisms for increased glycolysis in the hypertrophied rat heart. Hypertension. 2004;44(5):662‐667. 15466668 10.1161/01.HYP.0000144292.69599.0c

[71] Sutton‐McDowall ML , Gilchrist RB , Thompson JG . The pivotal role of glucose metabolism in determining oocyte developmental competence. Reproduction. 2010;139(4):685‐695. 20089664 10.1530/REP-09-0345

[72] Richardson MC , Ingamells S , Simonis CD et al Stimulation of lactate production in human granulosa cells by metformin and potential involvement of adenosine 5' monophosphate‐activated protein kinase. J Clin Endocrinol Metab. 2009;94(2):670‐677. 19001513 10.1210/jc.2008-2025

[73] Dunning KR , Cashman K , Russell DL , Thompson JG , Norman RJ , Robker RL . Beta‐oxidation is essential for mouse oocyte developmental competence and early embryo development. Biol Reprod. 2010;83(6):909‐918. 20686180 10.1095/biolreprod.110.084145

[74] Jiang X , Wang X . Cytochrome C‐mediated apoptosis. Annu Rev Biochem. 2004;73:87‐106. 15189137 10.1146/annurev.biochem.73.011303.073706

