# Supplementary figures and images for: Metformin protects against mouse oocyte apoptosis defects induced by arecoline
Source: Cell Prolif. 2020 Jun 17;53(7):e12809. doi: 10.1111/cpr.12809 (PMC7377942; doi:10.1111/cpr.12809)

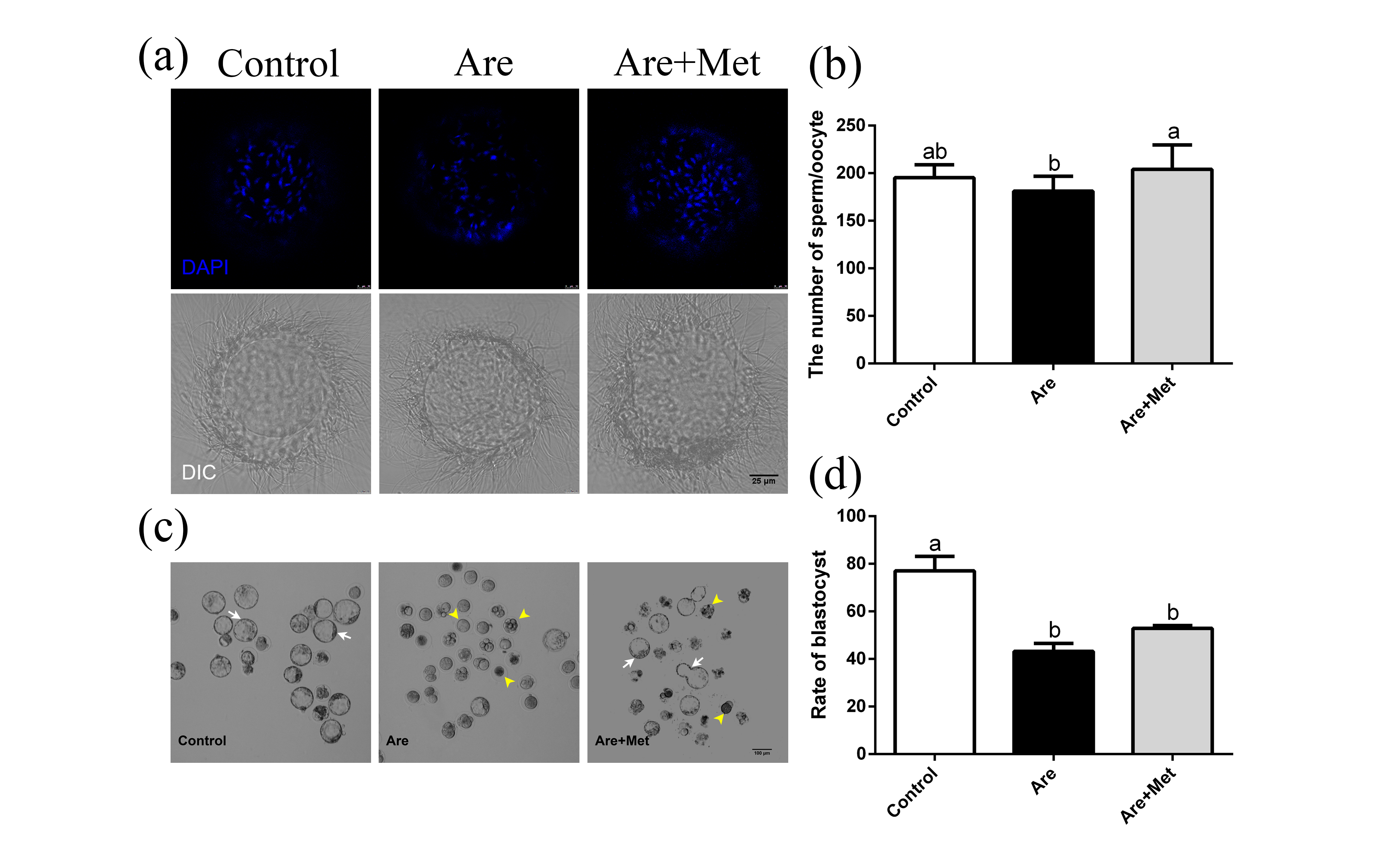

Supplement: Supplementary file 1 — Figure S1 [file CPR-53-e12809-s001.tif]
